# Association between Epstein-Barr virus and periodontitis: A meta-analysis

**DOI:** 10.1371/journal.pone.0258109

**Published:** 2021-10-07

**Authors:** Chaerita Maulani, Elza Ibrahim Auerkari, Sri Lelyati C. Masulili, Yuniarti Soeroso, Widayat Djoko Santoso, Lindawati S. Kusdhany

**Affiliations:** 1 Faculty of Dentistry, Doctoral Program, Universitas Indonesia, Jakarta, Indonesia; 2 Faculty of Dentistry, Department of Oral Biology, Universitas Indonesia, Jakarta, Indonesia; 3 Faculty of Dentistry, Department of Periodontology, Universitas Indonesia, Jakarta, Indonesia; 4 Faculty of Medicine, Department of Internal Medicine in Tropical Infection, Universitas Indonesia, Jakarta, Indonesia; 5 Faculty of Dentistry, Department of Prosthodontics, Universitas Indonesia, Jakarta, Indonesia; Klinikum der Johann Wolfgang Goethe-Universitat Frankfurt Klinik fur Nuklearmedizin, GERMANY

## Abstract

**Purpose:**

Previous studies have found that Epstein-Barr virus (EBV) is associated with periodontitis, though some controversy remains. This meta-analysis aimed to clarify and update the relationship between EBV and periodontitis as well as clinical parameters.

**Methods:**

A comprehensive search was conducted in the PubMed and Scopus databases in December 2020. Original data were extracted according to defined inclusion and exclusion criteria. Outcomes were analyzed, including overall odds ratios (ORs) and 95% confidence intervals (CIs). A random-effects model was used, and publication bias was assessed by Egger’s and Begg’s tests. Sensitivity analysis was used to evaluate the stability of the outcome.

**Results:**

Twenty-six studies were included in the present meta-analysis, involving 1354 periodontitis patients and 819 healthy controls. The included studies mostly showed high quality. The overall quantitative synthesis for the association between EBV and periodontitis was an increased odds ratio when subgingival EBV was detected OR = 7.069, 95% CI = 4.197–11.905, P<0.001). The results of subgroup analysis suggested that the association of EBV with periodontitis was significant in Asian, European, and American populations (P<0.001; P = 0.04; P = 0.003, respectively) but not in African populations (P = 0.29). Subgroup analysis by sample type showed that subgingival plaque (SgP), tissue and gingival crevicular fluid GCF were useful for EBV detection (P<0.001). EBV detection amplification methods included nested PCR, multiplex PCR and PCR (P<0.001; P = 0.05, P<0.001, respectively), but EBV detection by real-time PCR and loop-mediated isothermal amplification presented no significant result (P = 0.06; P = 0.3, respectively). For the clinical parameters of periodontitis, pocket depth (PD) and bleeding of probing (BOP) percentages were higher in the EBV-positive sites than in the EBV-negative sites (MD 0.47 [0.08, 0.85], P = 0.02; MD 19.45 [4.47, 34.43], P = 0.01).

**Conclusions:**

A high frequency of EBV detection is associated with an increased risk of periodontitis. The EBV association was particularly significant in all populations except in African populations. Subgigival plaque (SgP), tissue and GCF were not significantly different useful material for detecting EBV in periodontitis. Nested PCR and multiplex PCR are reliable methods for this purpose. In the presence of EBV, PD and BOP are reliable clinical parameters for gingival inflammation. However, some caution in such interpretation is justified due to heterogeneity among studies. A suggested extension could assess the parallel influence of other human herpesviruses.

## Introduction

Periodontitis is a periodontal condition involving progression beyond gingivitis to a chronic, destructive, irreversible inflammatory state, affecting the soft and hard tissues around the teeth. Severe periodontitis results in loss of attachment of the periodontium, loss of alveolar bone and subsequent loss of affected teeth [1]. The etiopathogenesis of periodontitis involves a complex interaction between specific bacterial pathogens and host cellular responses. Nevertheless, the onset and progression in individual cases are difficult to explain based on bacteria alone, and periodontal treatment is often not effective. Herpesviruses were added to the etiology of periodontitis in the late 1990s, highlighting the role of herpesvirus-bacteria interactions [2]. Many studies have been conducted since, and we sought to perform an updated meta-analysis to derive conclusions from previous and recent studies on periodontitis and its association with Epstein-Barr virus (EBV) that is a common representative of human herpesviruses. Other herpesviruses have also shown reported relatively common association to periodontitis, e.g. human cytomegalovirus (HCMV) and herpes simplex viruses (HSV). Further systemic impact is often virus-specific, so that for example EBV infection can promote cancer. In contrast, viral and bacterial coinfection may increase the severity of disease, which also applies to the reported impact of EBV in periodontitis.

EBV is a gamma-herpesvirus infecting more than 90% of adults worldwide [3]. As EBV has the ability to suppress host immunity, it is possible that EBV is a causative agent for periodontitis [4]. Some studies have indicated that EBV DNA is present in saliva, gingival crevicular fluid, subgingival plaque, and gingival tissue in periodontal pockets [5–7]. Further studies have found correlations between periodontitis and periodontal pocket depth [8,9]. Periodontitis in the previous classification consists of chronic periodontitis and aggressive periodontitis. However, difficulty in differentiating between aggressive periodontitis and chronic periodontitis arises when family history is not clear, and local factors are lacking after the patient’s initial treatment. Therefore, an American Academy of Periodontology task force suggested revision of the criteria that distinguish between aggressive and chronic periodontitis. Indeed, aggressive periodontitis and chronic periodontitis arenow called periodontitis in the newest periodontal disease classification [10,11].

A systematic review of six studies by Alzharani et al. on herpesviruses and aggressive periodontitis (AgP) reported increased EBV detection in AgP patients [12]. The meta-analysis of EBV and aggressive periodontitis by Fei Li et al. [13] showed a significant association (10 studies; OR = 6.11, 95% CI = 2.13–17.51, P = 0.0008), which was also indicated in the meta-analysis of EBV and chronic periodontitis by Ce Zhu et al. (OR = 5.74, 95% CI = 2.53–13.00, P<0.001) and by Gao et al. (OR = 6.586, 95% CI = 3.042–14.262, P<0.001) [14,15].

In a previous systematic review, periodontitis criteria were discussed with regard to aggressive periodontitis and/or chronic periodontitis. The present systematic review aimed to evaluate the association between EBV and periodontitis as a combination of chronic and aggressive periodontitis in systemically healthy patients.

## Materials and methods

This study was conducted based on Preferred Reporting Items for Systematic Review and Meta-Analysis (PRISMA) guidelines [16]. The research questions comprising the domains patients/population (P), exposure (E), comparator (C) and outcome (O) were as follows:

Population: periodontitis patients
Exposure: EBV positive
Comparator: EBV negative
Outcome: odds ratio for periodontitis events
The research question: What is the odds ratio for periodontitis in EBV-positive patients compared to EBV-negative patients?

### Search strategy

A systematic search was conducted through PubMed and Scopus up to December 10^th^, 2020. We independently searched all titles and abstracts using the following key terms: “Epstein-Barr virus” or “EBV” or “human herpesvirus 4” or HHV 4 AND “periodontitis”. The literature search was performed without any other restriction. Other available publications were identified from the reference lists of the selected literature.

### Eligibility criteria and study selection

The eligibility criteria of the selected studies included the following: designed as case-control or cross-sectional studies; target population of systemically healthy patients; comparing EBV detection in subjects with periodontitis and periodontally healthy controls; sampling from subgingival plaque, GCF or gingival tissue; EBV detection applying molecular methods such as polymerase chain reaction (PCR), nested PCR, multiplex, real-time PCR or loop-mediated isothermal amplification (LAMP); and published in English language only.

The exclusion criteria included in vitro and experimental studies, animal and cell studies, review papers, case reports, duplicate publications, no full article, sampling by saliva, and comparisons between the same diseased and healthy individuals.

### Data extraction

All potential reference studies were reviewed for study selection according to the above inclusion criteria. The following information was collected from each study: author, year, periodontitis type, country, sample size, mean age (year), sample type, sampling method, molecular analysis type and EBV prevalence.

The following clinical parameters to diagnose the case and control information were also collected: clinical attachment loss (CAL) and/or pocket depth (PD), radiographs, number of teeth, recorded periodontal clinical parameters, records of smoking, debridement and medication prior to sampling and EBV quantitative detection if any.

Periodontitis definition: Periodontitis according to the current classification eliminates the terms aggressive and chronic [11]. During data extraction, all cases of early-onset periodontitis, juvenile periodontitis, rapidly progressing periodontitis, and aggressive periodontitis were called periodontitis. Chronic marginal periodontitis, adult periodontitis, and chronic periodontitis are also referred to as periodontitis.

### Quality assessment

The quality of each study was assessed by independent reviewers using the Newcastle-Ottawa Scale (NOS) [17]. The NOS total quality scores range from 0 to 9 points. A higher score denotes better methodological quality. High-quality studies were considered at 7 points or higher. The quality of the included studies is presented in S1 Table of the Supporting Information.

### Data synthesis and analysis

The studies were summarized by the main findings in qualitative analysis. Then, meta-analysis was performed by combining the studies using a random-effects model. The association of EBV and periodontitis was estimated by odds ratios (95% confidence intervals), and the data are presented in a forest plot. Heterogeneity between studies was measured by *I*^*2*^. Subgroup analysis was performed by country, sample type and molecular detection method. The periodontal clinical parameters from some of the studies were assessed in this meta-analysis: clinical attachment loss (CAL), pocket depth (PD), gingival index (GI), plaque index (PI) and bleeding on probing percentage (BOP).

Additionally, sensitivity analysis was carried out excluding studies with poor quality. Egger’s asymmetry test, Begg’s test and funnel plots were used to analyze publication bias. Differences were considered statistically significant at P ≤ 0.05. Statistical analyses were performed in Review Manager (RevMan version 5.3 Copenhagen: The Nordic Cochrane Centre, The Cochrane Collaboration, 2014) and MedCalc® Statistical Software version 20 (MedCalc Software Ltd, Ostend, Belgium).

## Results

### Description of the Studies

**Fig 1:**
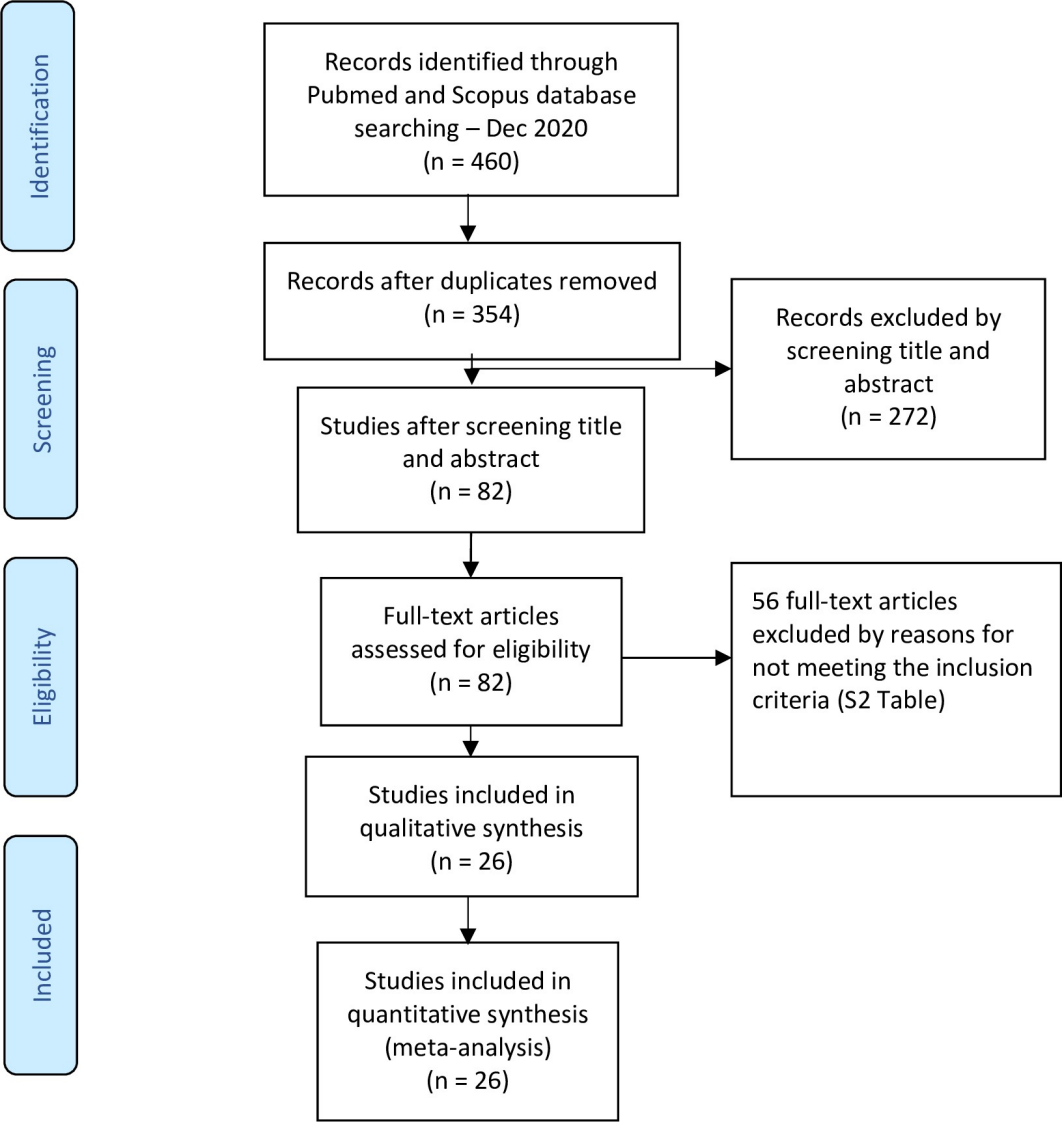
Flowchart of study selection.

The process of study selection is presented in Fig 1. Approximately 460 records were initially identified from PubMed and Scopus. After removing duplications, 354 records remained; 272 studies were excluded by screening titles and abstracts. The full texts of the remaining 82 articles were assessed in detail. In total, 56 articles were excluded for a range of reasons (see S2 Table in the Supporting Information). Thus, 26 eligible studies were included in the final meta-analysis (Fig 1).

The quality of the included studies is presented in S1 Table of the Supporting Information. All of the included studies were considered to be of high quality.

Of the included studies (Table 1), fourteen were from Asia [8,9,22–24,26,29,30,32–34,36,38,39], seven from Europe [6,7,20,21,25,28,31], three from America [18,19,27], and two from Africa [35,37]. The sample size varied from 25 [19] to 219 [24] patients. The mean age in the case groups ranged from 34.4 [28] to 57.4 [8] years for chronic periodontitis, from 15.4 [35] to 40.9 [7] for aggressive periodontitis and from 15.6 [35] to 52.9 [9] years for the control group. The sample type used in most of the studies was subgingival plaque; other studies used gingival tissue [7,19,38], and only one study used GCF [34]. For the sampling method, three studies used a single curette or paper point/filter strip [23,34,37], two studies used a combination [19,25], and others used pooled samples. Regarding molecular detection methods, twelve studies employed nested PCR [6–8,18,19,22–24,26,27,29,38], six real-time PCR [9,25,28,31,36,39], two multiplex PCR [34,37], five PCR [20,21,30,32,33] and one loop-mediated isothermal amplification [35].

**Table 1:** The characteristic of studies included in the meta-analysis.

Studies	Periodontitis types	Country	Sample size (cases/ control)	Mean age in years (cases/ control)	Material and methods			EBV prevalence (cases/control)
					Sample type	Sampling method	molecular detection method	
Contreras et al, 1999 [18]	AP	USA (American)	99/41	52.3/ 29	SgP	Pooled paper points	Nested	21/3
Contreras et al, 2000 [19]	AP, LJP	USA (American)	14/11	50	SgP& Tissue	Paper point and gingival biopsy	Nested	11/3
Saygun et al, 2002 [6]	CP	Turkey (European)	30/21	42.8/41.72	SgP	Pooled paper points	Nested	5/3
Yapar et al, 2003 [20]	AgP	Turkey (European)	17/16	24.05/24.12	SgP	Pooled curette samples	PCR	12/1
Saygun et al, 2004 [21]	AgP	Turkey (Europe)	18/16	24.1/24.1	SgP	Pooled curette samples	PCR	13/1
Wu et al, 2006 [22]	CP	China (Asian)	65/24	M 43.9; F 45.3/ M 37.3; F 36.3	SgP	Pooled paper points	Nested	43/4
Moghim et al, 2007 [23]	CP	Iran (Asian)	61/40	43/41.35	SgP	Single curette	Nested	37/1
Wu et al, 2007 [24]	CP	China (Asian)	143/76	M = 41.4; F = 42.5/ M = 38.8; F = 37.5	SgP	Pooled paper points	Nested	91/23
Sunde et al, 2008 [25]	CMP	Norway (European)	25/15	56/45	SgP	Curete and pooled paper points	Real-time	10/1
Rotola et al, 2008 [7]	CP, AgP	Italy (European)	CP 13; AgP11/13	CP 50.8; AgP 40.9/25.8	Gingival Biopsy	Single biopsy	Nested	12/1
Chalabi et al, 2008 [26]	CP	Iran (Asian)	61/40	42.9/40.7	SgP	Pooled curette samples	Nested	48/1
Imbronito et al, 2008 [27]	CP, AgP	Brazil (American)	CP 30; AgP 30/30	CP 42.7; AgP 27.3/ 28.1	SgP	Pooled paper points	Nested	24/0
Nibali et al, 2009 [28]	CP, AgP	UK (European)	CP 20; AgP 80; /40	34.4/50.3	SgP	Pooled curette samples	Real-time	6/4
Chalabi et al, 2010 [29]	CP	Iran (Asian)	40/40	40.9/42.0	SgP	Pooled curette samples	Nested	29/1
Sharma et al, 2012 [30]	CP, AgP	Indian (Asian)	CP 20; AgP 20/20	CP 42.53; AgP 29.65/ 36.52	SgP	Pooled curette samples	PCR	14/0
Stein et al, 2013 [31]	AgP	Germany (European)	65/65	35.4/40	SgP	Pooled paper points	Real-time	7/9
Kato et al, 2013 [8]	CP	Japan (Asian)	85/20	57.4/45.9	SgP	Pooled paper points	Nested	56/9
Joshi et al, 2015 [32]	CP	India (Asian)	100/100	NA	SgP	Pooled curette	PCR	21/6
Kato et al, 2015 [9]	CP	Japan (Asian)	25/13	54.2/52.9	SgP	Pooled paper points	Real-time	20/6
Sharma et al, 2015 [33]	AgP	India (Asian)	15/15	23.3/ 24.9	SgP	Pooled curette	PCR	6/1
Shah et al, 2016 [34]	CP	India (Asien)	40/20	40.7/ 29.3	GCF	Single filter strip	Multiplex	25/2
Elamin et al, 2017 [35]	AgP	Sudan (African)	17/17	15.4/15.6	SgP	Pooled paper points	LAMP	11/8
Srivastava et al, 2019 [36]	CP	India (Asian)	25/25	37.14/35.85	SgP	Pooled paper points	Real-time	19/4
Blankson et al, 2019 [37]	CP, AgP	Ghana (African)	LCP 5; GCP 7; AgP 9/10	LCP 40.6; GCP 46.3; AgP32.2/ NA	SgP	Single curette	Multiplex	1/0
Yu et al, 2020 [38]	CP, AgP	China (Asian)	CP 59; AgP 57/43	CP 48.53; AgP 29.74/ 28.81	Gingival tissue	Surgery	Nested & Real-time	53/5
Singhal et al, 2020 [39]	CP	India (Asian)	48/48	34.96/31.19	SgP	Pooled curette	Real-time	9/0

AgP = aggressive periodontitis; AP = adult periodontitis; CMP: Chronic marginalis periodontitis; EBV = Epstein-Barr virus; F = female; GAgP = generalized aggressive periodontitis; GCF = gingival crevicular fluid; GCP = generalized chronic periodontitis; LAgP = localized generalized periodontitis; LAMP = loop-mediated isothermal amplification; LCP = localized chronic periodontitis; LJP = localized juvenile periodontitis; M = male; NA = not available; PCR = polymerase chain reaction; SgP = subgingival plaque.

The clinical parameters assessed in the studies and confounding factors were evaluated (see S2 File in the Supporting Information). Approximately 42% of the studies used CAL, PD and radiographs to determine case and control groups, while 31% used CAL and PD without radiographs. Two studies (8%) mentioned that cases and controls were determined based only on periodontal classification by the American Academy of Periodontology. Twenty-three percent of the studies required at least 20 teeth for the included subjects; others only required ≥ 14 teeth (7.7%), ≥10 teeth (3.8%) or ≥ 9 teeth (7.7%), and 57.7% did not mention such a requirement. Smoking status was considered in 46.2% of the studies, though 53.8% did not mention details about smoking. Before samples were taken, some studies required no periodontal treatment (debridement) for the included subject (69.23%), whereas other studies required initial treatment before sampling (11.54%); 19.23% of the studies had no available data. The duration of no periodontal treatment was 6 months, 3 months or 12 months (38.5%, 26.9% and 3.8%, respectively). The duration of antibiotic restriction in the studies varied between 6, 3 and 2 months (42.3%, 38.5% and 3.8%, respectively). For the remaining 15.4% of the studies, no data on antibiotic provisions were available. Periodontitis-related clinical parameters were recorded in 80.8% of the studies, and no data were recorded in the remaining 19.2%. The most prevalent periodontitis-related clinical parameters were PD, CAL, GI, PI and BOP, which were recorded in 73.1%, 69.2%, 46.2% 42.3%, 38.5% of the studies, respectively. EBV quantitative detection was recorded from six studies, each of them using real-time PCR analysis methods [9,25,28,31,36,39].

### Quantitative synthesis

**Fig 2:**
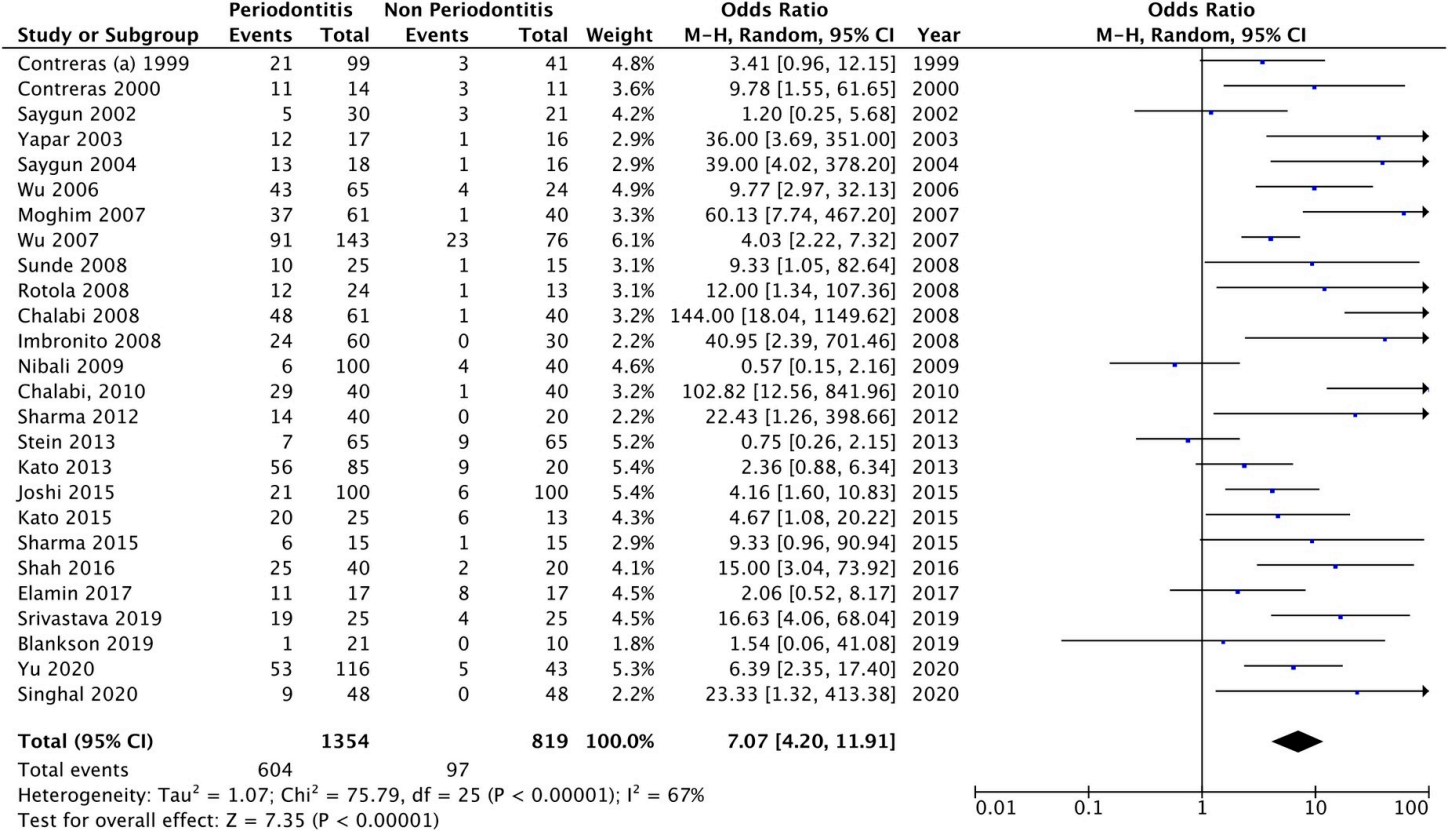
Forest plot analysis for the association between EBV and periodontitis.

**Fig 3:**
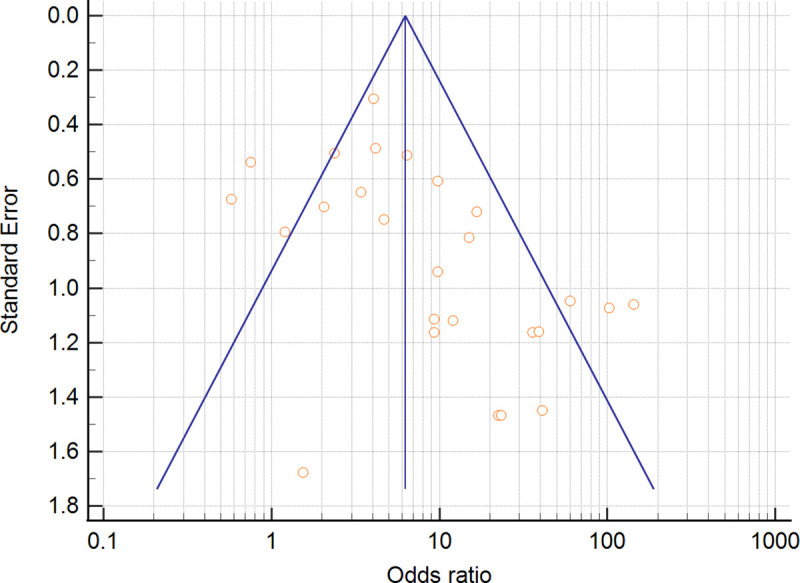
Funnel-plot analysis of the 26 studies in association of EBV detection and periodontitis.

The relationship between the risk of periodontitis and EBV detection was assessed in all included studies (n = 26), comprising 1354 periodontitis patients and 819 periodontally healthy controls. The overall results based on the random-effects model showed a significant association between EBV and the risk of chronic periodontitis (OR = 7.069, 95% CI = 4.197–11.905) (Fig 2). The random-effect model was used to generalize the results since the studies were not equivalent. Among the examined studies, 22 reported positive ORs, which ranged from 2.06 [35] to 144.00 [26], while only four [6,28,31,37] revealed no increased odds ratios. The study by Wu et al. [24] presented high weight (6.1%) in the meta-analysis. The heterogeneity test was 1.07, and the test for overall effect was *Z* = 7.35; *I*^*2*^ = 67% (P<0.0001), indicating significant heterogeneity. A funnel plot of the association between EBV and the risk of chronic periodontitis is presented in Fig 3.

The mean differences in periodontal parameters from subjects with periodontitis and EBV detection were analyzed. The results are presented separately for each periodontal parameter. The mean difference (MD), with a confidence interval of 95%, was calculated.

### Subgroup analysis

Subgroup analysis by country of origin showed that EBV was significantly associated with an increased risk of periodontitis in Asian, European and American populations (OR = 10.293, 95% CI = 5.624–18.837, P<0.001; OR = 4.039, 95% CI = 1.069–15.265 P = 0.040 and OR = 7,370, 95% CI = 2.017–26.934, P = 0.003, respectively). The African subgroup included two studies and was not associated with an increased risk of periodontitis (P = 0.29). The test for country subgroup showed no difference between groups (*I*^*2*^ = 50.4%, P = 0.11), suggesting that country of origin did not significantly modify the effect of EBV detection on the risk of periodontitis. There was also substantial heterogeneity within the Asian and European subgroups (*I*^*2*^ = 61.69%, P = 0.0012; *I*^*2*^ = 76.29%, P = 0.0003, respectively), whereas no heterogeneity was found for the American or African subgroup (*I*^*2*^ = 33.0%, P = 0.22; *I*^*2*^ = 0%, P = 0.871)

Subgroup analysis by sample type indicated that EBV detected using SgP, tissue and GCF was associated with periodontitis (OR = 6.829, 95% CI = 3.727–12.514, P<0.001; OR = 7.585, 95% CI = 3.353–17.161, P<0.001; OR = 15.00, 95% CI = 3.04–73.92, P = 0.0009, respectively). EBV detection by GCF only occurred in one study [34]; therefore, the heterogeneity test was not performed for the GCF subgroup. The test for subgroup differences indicated no significance (*I*^*2*^ = 0%, P = 0.66), suggesting that the method of obtaining samples did not modify the effect of EBV detection on the risk of periodontitis.

Subgroup analysis according to method demonstrated that EBV detection by nested PCR, multiplex PCR and PCR (OR = 9.125, 95% CI = 4.385–18.993, P<0.001; OR = 7.692, 95% CI = 1.007–58.745, P = 0.049; OR = 11.519, 95% CI = 4.005–33.131, P<0.001) was significantly associated with periodontitis. Subgroup analysis for real-time PCR and LAMP methods showed no significant association with EBV detection in periodontitis (OR = 3,629, 95% CI = 0.970–13.582, P = 0.056; OR = 2.06, 95% CI = 0.52–8.17, P = 0.30). As the LAMP method was only used in one study, the heterogeneity test was not conducted for this subgroup. The test for subgroup differences indicated no statistically significant subgroup effect (*I*^*2*^ = 25.1%, P = 0.25).

### Clinical parameter analysis

**Fig 4:**
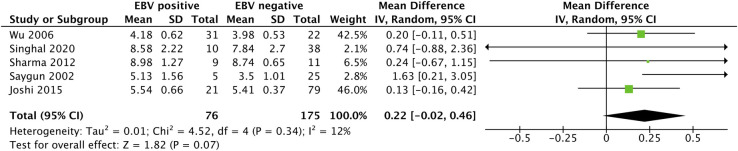
Forest plot analysis for the association between clinical attachment loss in periodontitis patients and EBV detection.

The clinical parameters of periodontitis are presented separately for each periodontal parameter. Five studies were included in the quantitative analysis of CAL [6,22,30,32,39], as illustrated in Fig 4. EBV-positive subjects (n = 76) had a greater mean CAL than those who were EBV negative (n = 175), but the difference was not significant (MD 0.22 [-0.02, 0.46]; P *=* 0.07), and no significant heterogeneity was found among the articles (*I*^*2*^ = 12%; P = 0.34).

**Fig 5:**
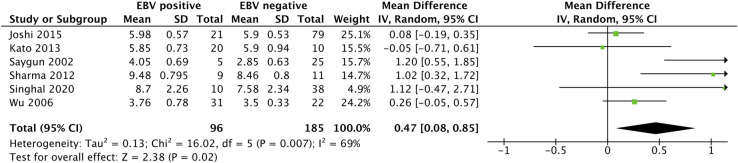
Forest plot analysis for the association between pocket depth in periodontitis patients and EBV detection.

PD was analyzed in six studies [6,8,22,30,32,39], as depicted in Fig 5. Although EBV-positive subjects (n = 96) had a mean PD that was greater than that of EBV-negative subjects (n = 185) (MD 0.47 [0.08, 0.85]; P *=* 0.02), there was considerable heterogeneity among the articles (*I*^*2*^ = 69%; P = 0.007).

**Fig 6:**
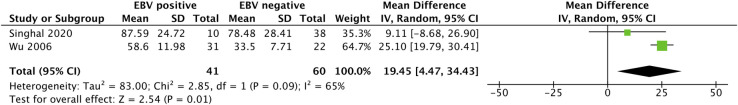
Forest plot analysis for the association between bleeding on probing (%) in periodontitis patients and EBV detection.

We found only two studies [22,39] including quantitative analysis of the percentage of BOP, as shown in Fig 6. EBV-positive subjects (n = 41) had a significantly higher percentage of BOP than EBV-negative subjects (n = 60) (MD 19.45 [4.47, 34.43]; P *=* 0.01), with no significant heterogeneity between the articles (*I*^*2*^ = 65%; P = 0.09).

**Fig 7:**
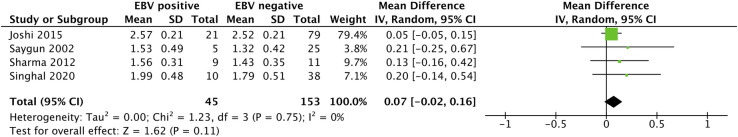
Forest plot analysis for the association between gingival index in periodontitis patients and EBV detection.

**Fig 8:**
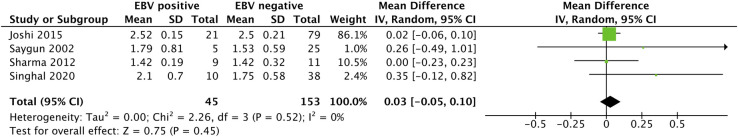
Forest plot analysis for the association between plaque index in periodontitis patients and EBV detection.

GI and PI were analyzed in four studies [6,30,32,39], as shown in Figs 7 and 8, respectively. EBV-positive subjects (n = 45) had a mean difference in GI and PI than those who were EBV negative (n = 185) (MD 0.07 [-0.02, 0.16]; P *=* 0.11; MD 0.03 [-0.05, 0.10]; P = 0.45). There was no heterogeneity among the articles regarding GI and PI analyses (*I*^*2*^ = 0%; P = 0.11; *I*^*2*^ = 0%; P = 0.45, respectively).

### Sensitivity analysis

To evaluate the stability of the pooled results, one study at a time was omitted from the meta-analysis, and the pooled result was consistent when any single study was omitted. Indeed, no single study changed the pooled ORs significantly in the overall meta-analysis, suggesting that the results were statistically stable and reliable.

### Publication bias

Publication bias was evaluated by Egger’s funnel plot asymmetry test and Begg’s test. The results are summarized in Table 2. Publication bias was apparent in the overall meta-analysis results (Egger’s test P = 0.0009; Begg’s test P = 0.0023). Significant publication bias was detected in all country subgroup analyses: Asian (Egger’s test P = 0.0015; Begg’s test 0.0186), European (Egger’s test P *=* 0.002; Begg’s test P = 0.004), American (Egger’s test: P = 0.049) and African (Egger’s test: P< 0001) populations. Publication bias was also found in sampling type subgroups SgP (Egger’s test P = 0.0019) and tissue (Egger’s test P = 0.017). For the subgroup molecular detection method, publication bias was found for nested PCR (Egger’s test P = 0.002; Begg’s test P = 0.028), subgroup multiplex PCR (Egger’s test P<0.0001) and PCR (Egger’s test: P = 0.041).

**Table 2:** Overall and subgroup analysis result.

Overall and subgroup analysis	Number of studies	Pooled OR	95% CI	*P*	Heterogeneity test			Publication bias (*P*)	
					*Q*	*P*	*I*^*2*^, %	Egger test	Begg test
Total	26	7.069	4.197–11.905	<0.001	75.788	<0.001	67.01	2.023	0.317
Country									
Asian	14	10.293	5.624–18.837	<0.001	33.929	0.001	61.69	2.643	0.473
European	7	4.039	1.069–15.2965	0.040	25.309	0.0003	76.29	5.861	0.905
American	3	7.370	2.017–26.934	0.003	2.991	0.224	33.12	3.171	1.000
African	2	1.974	0.554–7.029	0.294	0.026	0.871	0.00	-0.302	-1.000
Test group difference					6.04	0.11	50.4		
Sample type									
SgP	22	6.829	3.727–12.514	<0.001	72.775	<0.001	71.14	2.033	0.247
Tissue	3	7.585	3.353–17.161	<0.001	0.3535	0.838	0.00	1.020	1.000
GCF	1	15.00	3.04–73.92	0.0009	-	-	-	-	-
Test group difference			0.82	0.66	0				
Molecular detection methods									
Nested	12	9.125	4.385–18.993	<0.001	36.438	0.0001	69.81	2.476	0.485
Real-time	6	3.629	0.970–13.582	0.056	21.443	0.0007	76.68	4.211	0.600
Multiplex	2	7.692	1.007–58.745	0.049	1.495	0.221	33.12	-2.641	-1.000
PCR	5	11.519	4.005–33.131	<0.001	5.912	0.206	32.34	2.273	0.200
LAMP	1	2.06	0.52–8.17	0.30	-	-	-	-	-
Test group difference					5.34	0.25	25.1		

SgP = subgingival plaque, GCF = gingival crevicular fluid, OR = odds ratio, PCR = polymerase chain reaction.

## Discussion

There was no distinction between chronic and aggressive periodontitis in the 2017 classification system. This is because there was little evidence from biological studies that chronic and aggressive periodontitis were separate entities but rather were variations along a spectrum of the same disease process [40]. However, certain indicators that mark aggressive periodontitis still exist in recent classifications, such as molar and incisor patterns, especially in classical localized aggressive periodontitis [41]. Aggressive periodontitis seems to have a closer connection with herpesviruses, such as little plaque formation at sites with rapid and severe periodontal destruction, which cannot be explained solely by the roles of bacteria alone. This phenomenon might be better explained by herpesvirus such as EBV infection as a factor contributing to the change between active and latent periods [13,42]. This systematic review evaluated the association of EBV and periodontitis as an entity between chronic periodontitis and aggressive periodontitis according to the new classification, and only four of the included articles were from the new classification period. In the previous classification, chronic periodontitis was also called adult periodontitis or marginal periodontitis, while aggressive periodontitis was also called juvenile periodontitis or rapidly progressive periodontitis.

Previous review and meta-analysis studies have summarized published findings between EBV and chronic periodontitis and found a significant relationship [13–15]. We attempted to update the latest research and carry out a deeper analysis of periodontal clinical parameters. Twenty-six studies were included in the current meta-analysis. In twelve of them, EBV DNA was detected in more than 50% of infected sites [8,9,19–24,26,29,34,36], while three studies reported a very low prevalence of EBV in periodontitis sites [28,31,37]. All the studies presented high detection of EBV in periodontitis sites compared to in control groups, except for one study, which showed otherwise [31].

Most of the included studies had clear criteria for determination of cases and controls using one or more clinical parameter criteria, such as CAL or PD, but two studies did not mention the specific criteria applied. Nevertheless, we included them in our analyses because they mentioned defining cases and controls by the AAP classification [43]. CAL most likely involves a measurement error in the initial stage of periodontitis, but as disease severity increases, CAL can identify periodontitis with good accuracy [41].

Regarding signs of inflammation, some studies recorded bleeding on probing (BOP) [8,25–27,36,38], gingival index (GI) [32,39], or inflammation signs [9,19,21,22,24,36,44]. Overall, gingival inflammation cannot simultaneously be defined as a case of gingivitis because a patient with a history of periodontitis with gingival inflammation still has periodontitis [44]; therefore, the control group should be patients with no signs of inflammation or inflammation without a history of periodontitis/periodontal treatment. One study used gingivitis as a control instead of healthy periodontal tissue [18].

Fifty percent of the studies did not mention radiographs for determining cases and controls, while 42.3% required radiographs. Perhaps this is because periodontitis in the initial stage can be determined by CAL if radiographic bone loss is not available [41]. Among the included studies, the number of remaining teeth was recorded in 42.3%, whereas 57.7% had no available data. The minimal number of teeth recorded was ≥ 9. The number of teeth as a percentage of teeth present and the distribution of teeth have been used to define cases in the current periodontal classification system. However, if the most affected teeth in the dentition are lost, the severity of periodontitis may actually decrease, which is why the minimal number of teeth is an important factor for determining periodontitis cases [41].

Confounding factors were also taken into consideration in most of the studies, such as smoking, periodontal treatment, and antibiotic therapy before sampling. One study on gut microbiota found that antibiotics influence the microbiota by reducing diversity, though patients recovered to the baseline state within a few weeks and up to 2 or 6 months [45]. In 46.2% of studies, patients who smoked were recorded or excluded; no data were available for the remaining studies. Smoking is an important risk factor that increases progression to periodontitis [44]. In vitro experiments have also shown that cigarette smoke extracts promote EBV replication [46]. Thus, smoking is an important confounding factor in the association between EBV and periodontitis.

Approximately sixty-nine percent of the studies allowed no initial therapy of the included subjects before sampling, while 11.5% did not mention this requirement. The duration of no periodontal treatment was between 3 months and 12 months. Herpesviruses reside in periodontal inflammatory cells, and a reduction in gingival inflammation decreases the herpesvirus copy count [2]; therefore, most of the studies excluded subjects with initial therapy. Regardless, 19.2% of the studies required initial treatment. The reasons were because gingival tissue, which is harvested during periodontal surgery, was used and the subjects need initial therapy before surgery [7,38].

In the current meta-analysis, EBV detection was significantly associated with an increased risk of chronic periodontitis (OR = 7.069 95% CI = 4.197–11.905; P<0.001). Nevertheless, significant heterogeneity (*I*^*2*^ = 67.01%, P<0.0001) was found between the studies included in the quantitative synthesis, which was slightly higher than the odds ratio in a previous meta-analysis [15] (OR 6.199, 95% CI = 3.119–12.319) that showed heterogeneity (*I*^*2*^ = 74.3%, P*<*0.001).

Subgroup analysis was performed for country of origin, sample type and molecular detection methods. For country of origin, Asian, European and American subgroups exhibited a significant association between EBV detection and the risk of periodontitis, as in a previous study [15]. The largest odds ratio was found for the Asian subgroup, American subgroup, and European subgroup. The subgroup analysis also detected large heterogeneity, except for the American subgroup (*I*^*2*^ = 33.2, P = 0.224). The African subgroup was reported in two studies, with no significant association between EBV detection and the risk of periodontitis (P = 0.871). Publication bias was also found to be significant for all countries of origin. However, no test subgroup difference was observed for country of origin (*I*^*2*^ = 50.4%, P = 0.11), meaning that country of origin does not modify the effect of EBV detection on the risk of periodontitis. Additionally, there was substantial unexplained heterogeneity in some of the subgroups (Asian: *I*^*2*^ = 61.69%; European: *I*^*2*^ = 76.29%). Therefore, the validity of EBV detection for the risk of periodontitis in these subgroups is uncertain.

With respect to the subgroup sample type, SgP and tissue showed significant results (OR = 6.829, 95% CI 3.727–12.514; P<0.001, OR = 7.585, 95% CI 3.353–17.161; P<0.001). Heterogeneity was found only in the SgP subgroup, with none in the tissue subgroup (*I*^*2*^ = 71.17, P = <0.001; *I*^*2*^ = 0%, P = 0.35, respectively). As there were insufficient data for the GCF group, which was used in only one study, the validity of the results for this subgroup is uncertain.

In the subgroup molecular detection methods, a significant result was found for PCR, with the highest OR, followed by nested PCR and multiplex PCR (OR 11.519; 95% CI 4.005–33.131; P<0.001; OR = 9.125 95% CI 4.385–18.993, P < 0.001; OR = 7.692; 95% CI = 1.007–58.745; P = 0.049, respectively). Real-time detection (OR = 3.629; 95% CI = 0.970–13.582; P = 0.056) and LAMP showed no association between EBV detection and the risk of periodontitis. There were also insufficient data for the LAMP subgroup because it was only applied in one study. The heterogeneity value was large for real-time PCR and nested PCR (*I*^*2*^ = 76.68%, P = 0.0007; *I*^*2*^ = 69.81%, P = 0.0001, respectively). Moreover, no heterogeneity was found for the multiplex PCR and PCR groups (*I*^*2*^ = 33.1%, P = 0.221; *I*^*2*^ = 32.34%, P = 0.206, respectively). The test for subgroup differences in PCR method indicated no significant subgroup effect *(I*^*2*^ = 25.1%, P = 0.25), suggesting that the molecular detection method does not alter the effect of EBV detection on the risk of periodontitis. However, as there was a smaller number of studies and subjects in the multiplex PCR and LAMP subgroups, the analysis may not have been able to detect subgroup differences. EBV is a ubiquitous and life-long persistent infection; thus, quantitative measurement of the EBV genome is necessary to distinguish between low-level EBV infection in healthy carriers and high-level EBV in disease. Real-time quantitative PCR is currently the main method for EBV viral load measurement, and one of the advantages is the elimination of post-PCR manipulation [47]. In this study, no significant result was found because a very low prevalence of EBV was detected in two studies [28,31]; hence, the result was not significant in this subgroup.

Polymerase chain reaction (PCR) is used as a diagnostic tool for multiple periodontal pathogens because it is an accurate, sensitive, and rapid assay, even though it is prone to error. The presence of the DNA polymerase inhibitor EDTA during sample collection as well as alcohol in the analytical process can alter the diagnostic potential of PCR [48,49]. Introduced by Notomi et al. [50], loop-mediated isothermal amplification (LAMP) has superior specificity, efficiency, and ease of management for bacterial and herpesvirus identification [51]. The higher odds ratios found in studies before year 2012 [20,21,23,26,27,29] compared to those published thereafter. The detection rate of EBV might depend on different reasons including methodological ones. Different PCR techniques used to identify EBV DNA. Nested PCR is highly sensitive methods and most of the studies used nested PCR found an increased amount of EBV DNA. The risk of overestimating the results and the high risk of cross-contamination within the assay procedure. The technique has lower specificity in comparison to newer assay techniques [31,47].

Meta-analysis of clinical parameters revealed higher PD in EBV-positive subjects than in EBV-negative subjects (MD 0.47 [0.08, 0.85] and a higher percentage of BOP in EBV-positive subjects than in EBV-negative subjects (MD 19.45 [4.47, 34,43]) (P *=* 0.02; P = 0.01, respectively). The higher PD in EBV detection was in line with a study stating that herpesviruses reside in periodontal inflammatory cells and act as initiators of periodontopathic bacteria upgrowth [18,52], increasing periodontal destruction.

Numerous studies have proposed the biological mechanism responsible for the suspected association of EBV with the etiopathogenesis of periodontitis. EBV exhibits latent and lytic phases that establish a persistent infection in the host. Several bacteria, such as *Porphyromonas gingivalis* and *Fusobacterium nucleatum*, have been associated with periodontitis [4,53]. Kato et al. [8,9] found greater coexistence of OR EBV DNA with *P*. *gingivalis* in deeper sites in chronic periodontitis patients than in those with shallow sites and healthy sites. The periodontal pathogens *P*. *gingivalis* and *F*. *nucleatum* produce high levels of butyric acid [54], and a recent study showed that the saliva of chronic periodontitis patients contains butyric acid at higher levels than in healthy controls [55]. Butyric acid may play a role in the initiation of EBV reactivation and contribute to the clinical progression of patients with periodontal disease by inducing lytic switch activator BZLF1 expression in EBV [53]. The immediate-early BZLF1 gene encodes ZEBRA, which induces the lytic replication cycle in latently infected B cells [56]. Another study indicated the mechanism of EBV infection, which correlates with the severity of chronic periodontitis. EBV-encoded latent membrane protein 1 (LMP1) induces interleukin-2 (IL-8) production in human gingival cells [57,58], and the presence of IL-8 as well as proinflammatory cytokine levels in the gingival crevicular fluid are closely associated with the severity of inflammation and periodontal destruction [59].

The results of this meta-analysis showed an association between EBV and periodontitis, which can be useful for periodontal therapy. According to Olivieri et al [60], initial therapy or nonsurgical procedures as standard procedures for periodontitis in deep periodontal pockets are not sufficiently effective to eliminate EBV from periodontal sites. These authors identified large amounts of infiltrated EBV-infected cells mostly overlapping with CD138+ plasma cells [60]. In contrast, Kato et al. [61] found that initial periodontal therapy was effective in reducing the coexistence of EBV and *P*. *gingivalis* in the subgingival plaque of periodontitis patients, though it could not completely eradicate it. Another study showed that EBV can be treated by scaling and root planning, antiseptic treatment, and antiviral systemic medication [2].

Publication bias was found in this meta-analysis. Many forms of publication bias, such as time-lag bias (due to delayed publication), outcome reporting bias and language bias, tend to have more effects on small sample sizes than on larger sample sizes. Therefore, studies with small sample sizes tend to have larger and more favorable effects than studies with larger sample sizes, which compromises the validity of a systematic review and meta-analysis [62]. Most of the studies included in this systematic review were small in sample size, which may lead to exaggeration of the effect. The heterogeneity found between the studies in this meta-analysis might also affect the asymmetry of funnel plots.

Several limitations in this meta-analysis should be considered. First, substantial heterogeneity between the studies included in the quantitative synthesis for EBV was detected. The origin of the heterogeneity was not indicated by the data even after further subgroup analysis. A possible contributing reason could be the impact by undetected other herpesviruses such as human cytomegalovirus (HCMV) and herpes simplex viruses (HSV), with reported common association particularly to rapidly progressing (aggressive) periodontitis [63]. Second, only one study involved GCF and LAMP subgroups; only two studies involved African, multiplex PCR and BOP subgroups, possibly with insufficient power to test for a significant association. Third, publication bias was found in almost every subgroup, and thus the results for EBV and risk of periodontitis might be inconclusive. Last, language bias may exist since only studies published in the English language were retrieved and analyzed.

## Conclusions

A meta-analysis based on 26 studies involving 1354 patients with periodontitis and 819 healthy controls suggested that EBV is associated with an increased risk of periodontitis. This association was found in individuals of Asian, European, and American origin. However, as publication bias was found, the results must be interpreted cautiously. Subgroup analysis for sampling type showed a significant association for detection in subgingival plaque, tissue and GCF. Nonetheless, subgroup comparisons within GCF could not be established because of a lack of studies using GCF. The subgroup molecular detection method showed significant correlation of EBV and risk of periodontitis for nested, multiplex PCR and PCR but not real-time PCR and LAMP. No significant publication bias was found for multiplex PCR, but there was considerable publication bias for nested PCR and real-time PCR. The test for subgroup differences showed no difference between all subgroups, suggesting that country origin, sampling type and molecular detection methods do not modify the effect of EBV detection on the risk of periodontitis. This study provides a better understanding of the association between EBV and periodontitis. In the future, more studies are needed, as are data from various countries, especially Africa, with limited studies in this systematic review. EBV detection in saliva and the risk of periodontitis, which was not discussed in this study, should also be assessed. In addition, an extension is suggested to include the parallel contribution by other relatively common herpesviruses, in particular HCMV and HSV.

## Supporting information

S1 Table Quality score assessment of the included study. (DOCX)

S2 Table Articles of the excluded study with reasons. (DOCX)

S1 File PRISMA checklist. (PDF)

S2 File Clinical parameter to diagnose and recap. (DOCX)
