# Supplementary material for: Association between Epstein-Barr virus and periodontitis: A meta-analysis
Source: PLoS One. 2021 Oct 7;16(10):e0258109. doi: 10.1371/journal.pone.0258109 (PMC8496828; doi:10.1371/journal.pone.0258109)
Supplement: S1 Table — (DOCX) [file pone.0258109.s001.docx]

**Supplementary Table 1 Quality score assessment of the studies**

The quality of each studies was independently assessed using the Newcastle-Ottawa Scale (NOS). Total quality score was range from 0 point to 9 points. A higher score manifested better methodological quality. Studies with 7-9 considered to be high quality and study 4-6 points considered to be high risk quality and 0-3 very high risk of bias.

Well GA, Shea B, O’Connell D, Peterson J, Welch V, M Losos, et al (2000) The Newcastle-Ottawa Scale (NOS) for assessing the quality of nonrandomized studies in meta-analyses. Available at <http://www.ohri.ca/programs/clinical_epidemiology/nosgen.pdf>

| **Studies** | **Quality assessment Newcastle-Ottawa Scale (NOS)** | | | |
| --- | --- | --- | --- | --- |
|  | **Total** | **Selection** | **Comparability** | **Exposure/ Outcome** |
| Contreras, 1999 | 7 | 4 | 1 | 2 |
| Contreras, 2000 | 7 | 4 | 1 | 2 |
| Saygun, 2002 | 7 | 4 | 1 | 2 |
| Yapar, 2003 | 7 | 4 | 1 | 2 |
| Saygun, 2004 | 7 | 4 | 1 | 2 |
| Wu, 2006 | 8 | 4 | 2 | 2 |
| Moghim, 2007 | 7 | 4 | 1 | 2 |
| Wu, 2007 | 8 | 4 | 2 | 2 |
| Sunde, 2008 | 7 | 4 | 1 | 2 |
| Rotola, 2008 | 7 | 4 | 1 | 2 |
| Chalabi, 2008 | 7 | 4 | 1 | 2 |
| Imbronito, 2008 | 8 | 4 | 2 | 2 |
| Nibali, 2009 | 8 | 4 | 2 | 2 |
| Chalabi, 2010 | 7 | 4 | 1 | 2 |
| Sharma, 2012 | 7 | 4 | 1 | 2 |
| Stein 2013 | 8 | 4 | 2 | 2 |
| Kato, 2013 | 7 | 4 | 1 | 2 |
| Joshi, 2015 | 7 | 4 | 1 | 2 |
| Kato, 2015 | 7 | 4 | 1 | 2 |
| Sharma, 2015 | 7 | 4 | 1 | 2 |
| Shah, 2016 | 7 | 4 | 1 | 2 |
| Elamin, 2017 | 8 | 4 | 2 | 2 |
| Srivastava, 2019 | 7 | 4 | 1 | 2 |
| Blankson, 2019 | 7 | 4 | 1 | 2 |
| Yu, 2020 | 8 | 4 | 2 | 2 |
| Singhal, 2020 | 8 | 4 | 2 | 2 |
