# Supplementary material for: Association between Epstein-Barr virus and periodontitis: A meta-analysis
Source: PLoS One. 2021 Oct 7;16(10):e0258109. doi: 10.1371/journal.pone.0258109 (PMC8496828; doi:10.1371/journal.pone.0258109)
Supplement: S2 Table — (DOCX) [file pone.0258109.s002.docx]

| **Supplementary Table 2. Articles of the Excluded Studies with Reasons** | | |  | |
| --- | --- | --- | --- | --- |
| **Studies** | **Title** | **Reasons** | |  |
| Amaliya A, Laine ML, Delanghe JR, Loos BG, Van Wijk AJ, Van der Velden U. 2015 | Java project on periodontal diseases: periodontal bone loss in relation to environmental and systemic conditions | No subject with healthy periodontal as control group | |  |
| Antipa C, Bleotu C, Grancea C, Rosu AO, Anton G, Ruta S. 2016 | Viral serological and molecular data on possible involvement of herpes viruses in periodontal disease | No subject with healthy periodontal as control group | |  |
| Baez CF, Savassi-Ribas F, Rocha WM, Almeida SG, Gonçalves MT, Guimarães MA, Cavalcanti SM, Varella RB. 2016 | Association of Epstein-Barr Virus (EBV) but Not Human Papillomavirus (HPV) with Gingivitis and/or Periodontitis in Transplanted Individuals | Subject with systemically disease | |  |
| Bilichodmath S, Mangalekar SB, Sharma DC, Prabhakar AK, Reddy SB, Kalburgi NB, Patil SR, Bhat K. 2009 | Herpesviruses in chronic and aggressive periodontitis patients in an Indian population | No subject with healthy periodontal as control group | |  |
| Botero JE, Parra B, Jaramillo A, Contreras A. 2007 | Subgingival Human Cytomegalovirus Correlates with Increased Clinical Periodontal Parameters and Bacterial Coinfection in Subgingival Human Cytomegalovirus Correlates with Increased Clinical Periodontal Parameters and Bacterial Coinfection in Periodontitis | HCMV only no EBV | |  |
| Contreras A, Slots J. 1996 | Mammalian viruses in human periodontitis | No subject with healthy periodontal as control | |  |
| Dawson DR 3rd, Wang C, Danaher RJ, Lin Y, Kryscio RJ, Jacob RJ, Miller CS. 2009 | Salivary levels of Epstein-Barr virus DNA correlate with subgingival levels, not severity of periodontitis. | Not meeting inclusion criteria, sampling by saliva | |  |
| Dawson DR, Wang C, Danaher RJ, Lin Y, Kryscio RJ, Jacob RJ, Miller CS. 2009 | Real-Time Polymerase Chain Reaction to Determine the Prevalence and Copy Number of Epstein-Barr Virus and Cytomegalovirus DNA in Subgingival Plaque at Individual Healthy and Periodontal Disease Sites | No subject with healthy periodontal as control | |  |
| Ding F, Feng XH, Meng HX, Zhao YB, Zhang L, Lu RF, Chen ZB. 2008 | Relationship between herpesviruses and periodontal pathogenic bacteria in subgingival plaque | Full article in Chinese | |  |
| Ding F, Meng HX, Li QQ, Zhao YB, Feng XH, Zhang L. 2010 | Effect of Periodontal Mechanical Treatment on Herpesviruses in Gingival Crevicular Fluid of Patients with Chronic Periodontitis | Full article in Chinese | |  |
| Eke PI. 2011 | High PCR copy-counts of periodontal pathogens in saliva are associated with periodontal disease status | Not meeting inclusion criteria, sampling by saliva | |  |
| Emecen-Huja P, Danaher RJ, Dawson DR 3rd, Wang C, Kryscio RJ, Ebersole JL, Miller CS. 2020 | Relationship Between Herpesviruses and Periodontal Disease Progression | Not meeting inclusion criteria, randomized clinical trial | |  |
| Idesawa M, Sugano N, Ikeda K, Oshikawa M, Takane M, Seki K, Ito K. 2004 | Detection of Epstein-Barr virus in saliva by real-time PCR | Not meeting inclusion criteria, sampling only by saliva | |  |
| Ikeda Y, Kato A, Imai K, Ogata Y. 2020 | Quantitative Changes of *P. gingivalis* and EBV DNA in Saliva before and after Ini- tial Periodontal Therapy in Chronic Periodontitis Patients | Not meeting inclusion criteria, sampling only by saliva | |  |
| Imbronito AV, Okuda OS, Maria de Freitas N, Moreira Lotufo RF, Nunes FD. 2008 | Detection of Epstein-Barr virus and human cytomegalovirus in blood and oral samples: comparison of three sampling methods | No subject with healthy periodontal as control | |  |
| J Slots 1, J J Kamma, C Sugar. 2003 | The herpesvirus-Porphyromonas gingivalis-periodontitis axis | No subject with healthy periodontal as control group | |  |
| Kamma JJ, Contreras A, Slots J. 2001 | Herpes viruses and periodontopathic bacteria in early-onset periodontitis. | Control site is at the same subject as case site | |  |
| Kato A, Ikeda Y, Imai K, Ogata Y. 2020 | Effects of Initial Periodontal Therapy on the Prevalence of Epstein-Barr Virus DNA and *Porphyromonas gingivalis* in Japanese Chronic Periodontitis Patients | Control site is at the same subject as case site | |  |
| Kazi MM, Bharadwaj R, Bhat K, Happy D. 2015 | Association of Herpes Viruses with Mild, Moderate and Severe Chronic Periodontitis | No subject with healthy periodontal as control group | |  |
| Khosropanah H, Karandish M, Ziaeyan M, Jamalidoust M. 2015 | Quantification of Epstein-Barr Virus and Human Cytomegalovirus in Chronic Periodontal Patients | No subject with healthy periodontal as control group | |  |
| Kazi, M. and R. Bharadwaj. 2017 | Role of herpesviruses in chronic periodontitis and their association with clinical parameters and in increasing severity of the disease | No specific criteria for case and control group | |  |
| Klemenc P, Skaleric U, Artnik B, Nograsek P, Marin J.2005 | Prevalence of some herpesviruses in gingival crevicular fluid | No data for EBV frequency | |  |
| Koike R, Nodomi K, Watanabe N, Ogata Y, Takeichi O, Takei M, Kaneko T, Tonogi M, Kotani AI, Imai K. 2020 | Butyric Acid in Saliva of Chronic Periodontitis Patients Induces Transcription of the EBV Lytic Switch Activator BZLF1: A Pilot Study. | Not meeting inclusion criteria, experimental study | |  |
| Kubar A, Saygun I, O ̈zdemir A, Yapar M, Slots J. 2005 | Real-time polymerase chain reaction quantification of human cytomegalovirus and Epstein-Barr virus in periodontal pockets and the adjacent gingiva of periodontitis lesions | No subject with healthy periodontal as control group | |  |
| Li Y, Zhang JC, Zhang YH. 2004 | The association between infection of Epstein-Barr virus and chronic periodontitis | Full article in Chinese | |  |
| Li X, Sun QF, Sun YD, Ge SH, Yang PS. 2011 | Quantitative detection of human cytomegalovirus in aggressive and chronic periodontitis lesions | Full article in Chinese | |  |
| Ling LJ, Ho CC, Wu CY, Chen YT, Hung SL. 2004 | Association between human herpesviruses and the severity of periodontitis | No periodontal health subject as control group | |  |
| Olivieri CV, Raybaud H, Tonoyan L, Abid S, Marsault R, Chevalier M, Doglio A, Vincent-Bugnas S. 2020 | Epstein-Barr virus-infected plasma cells in periodontitis lesions | No subject with healthy periodontal as control group | |  |
| Parra B, Slots J. 1996 | Detection of human viruses in periodontal pockets using polymerase chain reaction. | No specific criteria for case and control group | |  |
| Parthiban S, Ramakrishnan T, Shankarram V, Balakumar V, Rai R, Umasudhakar. 2015 | Estimation of Load of Herpes Viruses in GCF and its Impact on Clinical Parameters in Periodontitis | No information regarding the EBV detection frequency | |  |
| Passariello C, Gigola P, Testarelli L, Puttini M, Schippa S, Petti S. 2017 | Evaluation of microbiota associated with Herpesviruses in active sites of generalized aggressive periodontitis | Not meeting inclusion criteria, only aggressive periodontitis | |  |
| Ortiz PS, 2016 | Prevalencia de los herpes virus en pacientes con enfermedad periodontal crónica y enfermedad periodontal agresiva | Full article in Spanish | |  |
| Reichart PA. 1999 | Infektionen der Mundschleimhaut (Teil II). Bakterielle, mykotische und virale infektionen [Infections of the oral mucosa II. Bacterial, mycotic and viral infections | Full article in Germany | |  |
| Santangelo R, D'Ercole S, Graffeo R, Marchetti S, Deli G, Nacci A, Piccolomini R, Cattani P, Fadda G. 2004 | Bacterial and viral DNA in periodontal disease: a study using multiplex PCR | No full article | |  |
| Saygun I, Kubar A, Sahin S, Sener K, Slots J. 2008 | Quantitative analysis of association between herpesviruses and bacterial pathogens in periodontitis | No specific criteria for case and control group | |  |
| Saygun I, Kubar A, Ozdemir A, Slots J. 2005 | Periodontitis lesions are a source of salivary cytomegalovirus and Epstein-Barr virus | Not meeting inclusion criteria, sampling by saliva | |  |
| Saygun I, Nizam N, Keskiner I, Bal V, Kubar A, Açıkel C, Serdar M, Slots J. 2011 | Salivary infectious agents and periodontal disease status | Not meeting inclusion criteria, sampling by saliva | |  |
| Solomon SM, Filioreanu AM, Stelea CG, Grigoras SO, Sufaru IG, Maftei GA, Martu S, Scutariu MM, Popa C. 2018 | The Assesment of the Association Between Herpesviruses and Subgingival Bacterial Plaque by Real-time PCR Analysis | No specific criteria for case and control group | |  |
| Tiantian, M. and L. Xin. 2016 | Promotion of Porphyromonas gingivalis to viral disease | Full article in Chinese | |  |
| Ting M, Contreras A, Slots J. 2000 | Herpesvirus in localized juvenile periodontitis | Not meeting inclusion criteria, only aggressive periodontitis | |  |
| Vincent-Bugnas S, Vitale S, Mouline CC, Khaali W, Charbit Y, Mahler P, Prêcheur I, Hofman P, Maryanski JL, Doglio A. 2013 | EBV Infection Is Common in Gingival Epithelial Cells of the Periodontium and Worsens during Chronic Periodontitis | Not meet the inclusion criteria, cell study | |  |
| Watanabe N, Nodomi K, Koike R, Kato A, Takeichi O, Kotani AI, Kaneko T, Sakagami H, Takei M, Ogata Y, Sato S, Imai K. 2019 | EBV LMP1 in Gingival Epithelium Potentially Contributes to Human Chronic Periodontitis via Inducible IL8 Production | Not meeting inclusion criteria, cell study | |  |
| Watanabe SA, Correia-Silva Jde F, Horta MC, Costa JE, Gomez RS. 2007 | EBV-1 and HCMV in aggressive periodontitis in Brazilian patients | Not meeting inclusion criteria, only aggressive periodontitis | |  |
| Wu YM, Chen LL, Yan J, Sun WL, Gu ZY. 2005 | Infection frequency of Epstein-Barr virus in subgingival samples from patients with different periodontal status and its correlation with severity of periodontal lesion | Full article in Chinese | |  |
| Gao Z, Lv J, Wang M. 2017 | Epstein-Barr virus is associated with periodontal diseases: A meta-analysis based on 21 case-control studies | Review article | |  |
| Imai, K. and Y. Ogata. 2020 | How Does Epstein-Barr Virus Contribute to Chronic Periodontitis? | Review article | |  |
| Li F, Zhu C, Deng FY, Wong MCM, Lu HX, Feng XP. 2017 | Herpesviruses in etiopathogenesis of aggressive periodontitis: A meta-analysis based on case-control studies | Review article | |  |
| Chen C, Feng P, Slots J. 2020 | Herpesvirus-bacteria synergistic interaction in periodontitis | Review article | |  |
| Slots, J. and H. Slots. 2019 | Periodontal herpesvirus morbidity and treatment | Review article | |  |
| Tonoyan L, Chevalier M, Vincent-Bugnas S, Marsault R, Doglio A. 2019 | New Viral Facets in Oral Diseases: The EBV Paradox | Review article | |  |
| Zhu C, Li F, Wong MC, Feng XP, Lu HX, Xu W. 2015 | Association between Herpesviruses and Chronic Periodontitis: A Meta-Analysis Based on Case-Control Studies | Review article | |  |
