# Supplementary material for: Association between Epstein-Barr virus and periodontitis: A meta-analysis
Source: PLoS One. 2021 Oct 7;16(10):e0258109. doi: 10.1371/journal.pone.0258109 (PMC8496828; doi:10.1371/journal.pone.0258109)
Supplement: S2 File — (DOCX) [file pone.0258109.s004.docx]

Supplementary Table 1. Clinical parameter to diagnose

Supplementary Table 2. Recap variable to diagnose and graphics

| **Clinical parameter to diagnose case control** | **Percentage** | **Frequency (n=26)** |
| --- | --- | --- |
| CAL, PD, No Ro | 30,8 | 8 |
| CAL, No Ro | 19,2 | 5 |
| CAL, PD, Ro | 42,3 | 11 |
| Based on AAP Classiffication | 7,7 | 2 |
| Total | 100 | 26 |
|  |  |  |
| **Number of teeth** | **Percentage** | **Frequency (n=26)** |
| ≥ 20 teeth | 23,1 | 6 |
| ≥ 14 teeth | 7,7 | 2 |
| ≥ 10 teeth | 3,8 | 1 |
| ≥ 9 teeth | 7,7 | 2 |
| NA | 57,7 | 15 |
| Total | 100 | 26 |
|  |  |  |
| **Smoking status** | **Percentage** | **Frequency (n=26)** |
| Recorded/Excluded | 46,2 | 12 |
| NA | 53,8 | 14 |
| Total | 100 | 26 |
|  |  |  |
| Duration periodontal treatment and antibiotic before sampling | | |
| **Debridement** | **Percentage** | **Frequency (n=26)** |
| A. No, 3 months | 26,9 | 7 |
| B. No, 6 months | 38,5 | 10 |
| C. No, 12 months | 3,8 | 1 |
| D. Yes | 11,5 | 3 |
| E. NA | 19,2 | 5 |
| Total | 100 | 26 |
|  |  |  |
| **Antibiotic** | **Percentage** | **Frequency (n=26)** |
| A. No, 2 months | 3,8 | 1 |
| b. No, 3 months | 38,5 | 10 |
| C. No, 6 months | 42,3 | 11 |
| D. NA | 15,4 | 4 |
| Total | 100 | 26 |
|  |  |  |
| **Clinical parameter of periodontal** | **Percentage** | **Frequency (n=26)** |
| Recorded | 80,8 | 21 |
| NA | 19,2 | 5 |
| Total | 100 | 26 |
|  |  |  |
| **Types of recorded clinical parameter** | **Percentage** | **Frequency (n=26)** |
| PD | 73,1 | 19 |
| CAL | 69,2 | 18 |
| BOP | 38,5 | 10 |
| GI | 46,2 | 12 |
| PI | 42,3 | 11 |
| Clinical mobility | 7,7 | 2 |
| Signs of inflammation | 3,8 | 1 |
| Number of teeth | 3,8 | 1 |
| SBI | 3,8 | 1 |

Supplementary Table 3. Clinical Parameter

| **Table. 3 Clinical parameter Periodontal** | | | |  |  |  |
| --- | --- | --- | --- | --- | --- | --- |
| **CAL** | **EBV positive** | | | **EBV negative** | | |
|  | **Mean** | **SD** | **Total** | **Mean** | **SD** | **Total** |
| Joshi 2015 | 5,54 | 0,66 | 21 | 5,41 | 0,37 | 79 |
| Saygun 2002 | 5,13 | 1,56 | 5 | 3,5 | 1,01 | 25 |
| Sharma 2012 | 8,98 | 1,27 | 9 | 8,74 | 0,65 | 11 |
| Singhal 2020 | 8,5 | 2,22 | 10 | 7,84 | 2,7 | 38 |
| Wu 2006 | 4,18 | 0,62 | 31 | 3,98 | 0,53 | 22 |
|  |  |  |  |  |  |  |
| **PD** | **EBV positive** | | | **EBV negative** | | |
|  | **Mean** | **SD** | **Total** | **Mean** | **SD** | **Total** |
| Joshi 2015 | 5,98 | 0,57 | 21 | 5,9 | 0,53 | 79 |
| Saygun 2002 | 4,05 | 0,69 | 5 | 2,85 | 0,63 | 25 |
| Sharma 2012 | 9,48 | 0,795 | 9 | 8,46 | 0,8 | 11 |
| Singhal 2020 | 8,7 | 2,26 | 10 | 7,58 | 2,34 | 38 |
| Wu 2006 | 3,76 | 0,78 | 31 | 3,5 | 0,33 | 22 |
| Kato 2013 | 5,85 | 0,73 | 20 | 5,9 | 0,94 | 10 |
|  |  |  |  |  |  |  |
| **PI** | **EBV positive** | | | **EBV negative** | | |
|  | **Mean** | **SD** | **Total** | **Mean** | **SD** | **Total** |
| Joshi 2015 | 2,52 | 0,15 | 21 | 2,5 | 0,21 | 79 |
| Saygun 2002 | 1,79 | 0,81 | 5 | 1,53 | 0,59 | 25 |
| Sharma 2012 | 1,42 | 0,19 | 9 | 1,42 | 0,32 | 11 |
| Singhal 2020 | 2,1 | 0,7 | 10 | 1,75 | 0,58 | 38 |
|  |  |  |  |  |  |  |
| **GI** | **EBV positive** | | | **EBV negative** | | |
|  | **Mean** | **SD** | **Total** | **Mean** | **SD** | **Total** |
| Joshi 2015 | 2,57 | 0,21 | 21 | 2,52 | 0,21 | 79 |
| Saygun 2002 | 1,53 | 0,49 | 5 | 1,32 | 0,42 | 25 |
| Sharma 2012 | 1,56 | 0,31 | 9 | 1,43 | 0,35 | 11 |
| Singhal 2020 | 1,99 | 0,48 | 10 | 1,79 | 0,51 | 38 |
|  |  |  |  |  |  |  |
| **BOP %** | **EBV positive** | | | **EBV negative** | | |
|  | **Mean** | **SD** | **Total** | **Mean** | **SD** | **Total** |
| Singhal 2020 | 87,59 | 24,72 | 10 | 78,48 | 28,41 | 38 |
| Wu 2006 | 58,6 | 11,98 | 31 | 33,5 | 7,71 | 22 |
